# COVID-19: Racism Is Like That

**DOI:** 10.1089/heq.2020.0063

**Published:** 2020-10-19

**Authors:** Elizabeth A. Brown

**Affiliations:** Department of Health Professions, College of Health Professions, Medical University of South Carolina (MUSC), Charleston, South Carolina, USA.

**Keywords:** COVID-19, racism, African American

## Abstract

Coronavirus disease 2019 (COVID-19)'s impact has similar characteristics to racism and its effects. First, there is no known immunity to COVID-19 or racism. Second, we wear uncomfortable masks to protect us from the virus. Being black in America requires wearing an uncomfortable invisible mask, hiding anxiety and fear. Third, physical distancing is promoted to reduce COVID-19 transmission. With racism, physical distancing has occurred from the Atlantic Slave Trade to segregation and redlining. COVID-19 has punished communities of color, just like racism has. COVID-19 has suffocated America just like racism does to blacks. If America is tired of COVID-19, imagine how blacks feel.

## Introduction

I am a black woman born and raised in the south. My parents and all four of my grandparents were born and raised in the south. My mother was raised in the Atlanta public housing system and experienced the city's energy with Martin Luther King, Jr. and the fight for equality. My father was the son of sharecroppers, raised in a small rural town in Alabama and experienced racism firsthand as his high school began to integrate in the 1960s. Their stories opened a window into how blacks experienced racism in the big cities to the small rural towns of southern America.

**FIG. 1.**
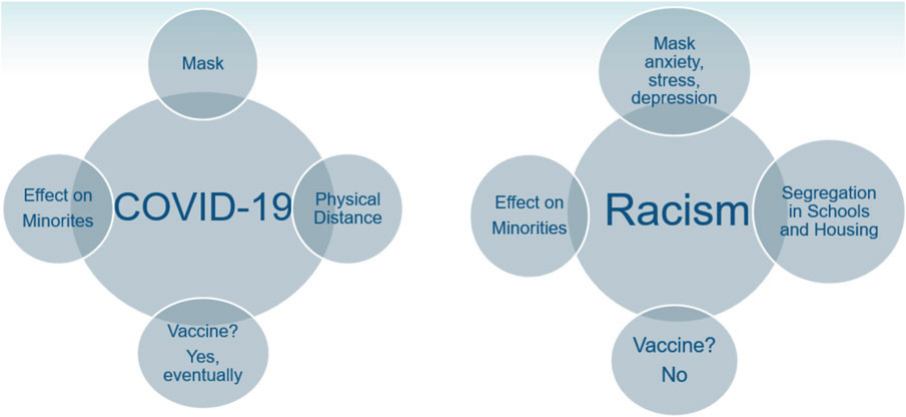
Similarities between COVID-19 and racism. Masks hide our faces and sometimes our emotions. Physical distancing protects us from virus transmission, but, in racism, physical distancing occurs with segregation and redlining. COVID-19 has had a substantial impact on minority communities. The same can be said for racism and its effect on minorities. Finally, there may be a cure for both, but will we see a cure for both COVID-19 and racism? COVID-19, coronavirus disease 2019.

Now, having more time to reflect on their stories as well as my own experiences in the south have led me an interesting realization as a black woman in the southern United States—coronavirus disease 2019 (COVID-19) may open a small window for white America to see how black Americans experience racism… at least just a little bit. Believe it or not, COVID-19 and racism have a lot in common (Fig. 1).

## Immunity

Currently, there is no known immunity for COVID-19. In other words, we do not know how the human body can (1) protect itself from getting the virus if we encounter it and (2) ward off the harmful effects of the virus. *Racism is like that.* We cannot protect our black and brown children from racism or ward off the effects of racism. To reach immunity where racism has no effect, we should follow the COVID-19 race to the cure. Just as we have (1) acknowledged COVID-19 is real and deadly and (2) put our energy in finding a cure, we should do the same with racism. Unfortunately, right now, no one has immunity from COVID-19, and black Americans do not have immunity from racism.

## Masks

In the age of COVID-19, public health experts advise us to wear masks for our protection. Many of us are not used to wearing masks and feel uncomfortable. Masks stifle our breathing, hide our smiles, and prevent us from showing our true selves. *Racism is like that.* Minorities, particularly black Americans, wear an invisible mask each day they open their eyes and face the world.^1^ The invisible masks that blacks wear hide the anger, hurt, fear, depression, anxiety, and even post-traumatic stress disorder (PTSD) we carry living in a society that does not value us because of something as frivolous as the color of our skin. Once COVID-19 ceases to exist, the protestors go home, and we move beyond this racial unrest, blacks will continue to wear their invisible masks. Under our masks, there is fear and anxiety from being treated differently when going for a walk or run in the neighborhood (Elijah McClain and Ahmaud Arbery) or being approached by the police (Sandra Bland, Eric Garner, Walter Scott, Breonna Taylor, and Rayshard Brooks). There is anger, hurt, and I dare to say PTSD when blacks *repeatedly* experience excessive, violent, and deadly outcomes for nonviolent offenses. With the recent murder of Mr. George Floyd, blacks grieve, yet again, the deaths of Sandra Bland, Tamir Rice, Walter Scott, Freddie Gray, Emmanuel 9, Philando Castille, Trayvon Martin, and so many countless others (#SayTheirNames). But, through the pain and agony, we wear our invisible mask, stifling our very souls in a shadow of anxiety.

## Physical Distance

Public health experts advise that we distance ourselves physically to decrease COVID-19 transmission. Experts tell us the ideal physical distance is approximately six to eight feet. When we stand in line in public places, we should adhere to the signs and put distance in between ourselves. *Racism is like that.* Blacks experienced physical distancing from our homelands (Atlantic Slave Trade). Blacks have experienced racial segregation since the day they arrived in the colonies. We were separated from plantation owners and overseers based on our race and color of our skin. Later, we experienced physical distancing in schools, restaurants, and residential communities (segregation and redlining). Now, we experience physical distancing in school districts and residential communities because of the disadvantages related to systemic racism combined with poor socioeconomic status. Here, in South Carolina, as probably with many states across the United States, we see poor outcomes for blacks in education,^2^ unemployment,^3^ and income.^4^ Physical distance for black Americans has been deeply ingrained in the social structure of the United States for >400 years. With the Atlantic Slave Trade, we were displaced and distanced from our original homelands, loved ones, native tongue, and cultural and religious norms. Black Americans have experienced physical distancing for 400+ years now.

## Impact on Minorities

Although COVID-19 has taken many lives, it has hit black and brown communities particularly hard.^5^ The virus, compounded with lower socioeconomic status, lack of health insurance, and a higher prevalence of underlying health conditions in blacks, has been too much of a burden to carry for minority communities. Even if minorities seek care for COVID-19, we have to consider many things, including how many have adequate access to a provider they trust, how many get the same quality treatment as their white counterparts, and how many can afford to take days off from work and lose income. Frankly put, COVID-19 has punished communities of color. *Racism is like that.* Racism is steeped in separating people by their race and skin color, punishing them because of this difference.

## Vaccine

We must patiently wait on clinical trials and research to prove a vaccine is safe and effective. In short, we do not have a vaccine for COVID-19, but we are hopeful we will have one soon. *Racism is like that.* We do not have a vaccine or cure for racism but hope to have it one day. Many blacks continue to ask, “When?” When will racism end? According to Ms. Roxane Gay, we will eventually have a COVID-19 vaccine; however, we will still be searching for a cure for racism.^6^ We will not find the cure for racism in a pharmaceutical laboratory but in the hands of Americans who (1) acknowledge there are racial disadvantages in the United States; (2) require a public national apology to descendants of African slaves in the United States; (3) ensure equal access to voting to change policies in various areas, specifically, education, employment, housing, and health insurance; (4) promote equal access to advantageous educational, financial, and housing opportunities; and (5) provide monetary reparations to those who are descendants of African slaves in the United States. Focusing on these areas of need can close gaps in racial disparities and formulate the cure for systemic racism in the United States. I dare say if we can find vaccines and cures for polio, tetanus, and other debilitating diseases, we can find a cure for racism.

## Conclusion

Dr. Robert Redfield recently said COVID-19 has “brought this nation to its knees.”^7^ Well, if you did not know already, America, so has racism. Racism brought minorities to their knees a long time ago. It is time we all stand up. The first way to do this is changing systems so that minorities (and other disadvantaged groups) have the same privileges, advantages, and opportunities as wealthy Americans in school systems, judicial systems, and financial systems. America, notably white America, if you are tired of COVID-19 and its effects, imagine how black Americans and minorities feel.

## Abbreviations Used

COVID-19: coronavirus disease 2019
PTSD: post-traumatic stress disorder
